# Influence of early hemodialysis on the septic acute kidney injury outcome^*^

**DOI:** 10.1590/1980-220X-REEUSP-2022-0109en

**Published:** 2022-11-07

**Authors:** Cléria Alves de Queiroz, Marcelo Rodrigues Bacci

**Affiliations:** 1 Centro Universitário São Francisco de Barreiras, Departamento de Unidade de Terapia Intensiva, Barreiras, BA, Brazil.; 2 Centro Universitário Faculdade de Medicina ABC, Departamento de Análises Clínicas, Santo André, SP, Brazil.

**Keywords:** Kidney Diseases, Sepsis, Renal Dialysis, Enfermedades Renales, Sepsis, Diálisis Renal, Nefropatias, Sepse, Diálise Renal

## Abstract

**Objective:**

to analyze the influence of early hemodialysis on the outcome of acute septic kidney injury.

**Method:**

this is an observational, analytical, prospective study with patients diagnosed with acute septic kidney injury on hemodialysis. A questionnaire for data collection was used as an instrument. We used the Shapiro-Wilk, nonparametric Kruskal-Wallis, Mann-Whitney U, Student t and chi-square tests for analysis.

**Results:**

of the 40 patients analyzed, 60% were male, with a mean age of 55 (±16.8) years, and length of hospital stay of 43 (±26.2) days. When separating patients undergoing early and late hemodialysis into two groups, an increase in serum creatinine (p = 0.001) was observed in those who underwent late hemodialysis, however, creatinine ≥ 4mg/dl is one of the characteristics of this group. In both groups, there was a high mortality: 62.5% (10) in the early hemodialysis group and 41.7% (10) in the late hemodialysis group, with vasopressor use (p = 0.001) being the main risk factor.

**Conclusion:**

early onset of hemodialysis in acute septic kidney injury based on KDIGO definitions did not influence the outcome. However, vasopressor use associated with hemodialysis in septic patients was a predictor of death.

## RESUMO

**Objetivo:**

analisar a influência da hemodiálise precoce no desfecho da lesão renal aguda séptica.

**Método:**

estudo observacional, analítico, prospectivo, com pacientes diagnosticados com lesão renal aguda séptica em hemodiálise. Foi utilizado como instrumento um questionário para coleta de dados. Utilizaram-se para análise os testes Shapiro-Wilk, o não paramétrico de Kruskal-Wallis, U de Mann-Whitney, t de Student e do Qui-Quadrado.

**Resultados:**

dos 40 pacientes analisados, 60% eram do sexo masculino, com média de 55 (±16,8) anos, e tempo de internação hospitalar de 43 (±26,2) dias. Ao separar em dois grupos, pacientes submetidos à hemodiálise precoce e à hemodiálise tardia, observou-se naqueles que realizaram tardiamente a hemodiálise um aumento de creatinina sérica (p = 0,001), entretanto a creatinina ≥ 4mg/dl é umas das características desse grupo. Em ambos os grupos, houve uma alta mortalidade: 62,5% (10) no grupo de hemodiálise precoce e 41,7% (10) no grupo de hemodiálise tardia, sendo o uso de vasopressor (p = 0,001) o principal fator de risco.

**Conclusão:**

o início precoce da hemodiálise na lesão renal aguda séptica com base nas definições do KDIGO não influenciou no desfecho. Contudo, o uso do vasopressor associado à hemodiálise em pacientes sépticos foi um fator preditor ao óbito.

## RESUMEN

**Objetivo:**

analizar la influencia de la hemodiálisis precoz en el desenlace del daño renal agudo séptico.

**Método:**

estudio observacional, analítico, prospectivo con pacientes diagnosticados de insuficiencia renal aguda séptica en hemodiálisis. Se utilizó un cuestionario como instrumento para la recolección de datos. Para el análisis se utilizaron las pruebas de Shapiro-Wilk, Kruskal-Wallis no paramétrica, U de Mann-Whitney, t de Student y Chi-Cuadrado.

**Resultados:**

de los 40 pacientes analizados, el 60% eran del sexo masculino, con una edad media de 55 (±16,8) años y una estancia hospitalaria de 43 (±26,2) días. Al separar a los pacientes en hemodiálisis temprana y tardía en dos grupos, se observó un aumento de la creatinina sérica (p = 0,001) en los que realizaron hemodiálisis tardía, sin embargo, la creatinina ≥ 4 mg/dl es una de las características de este grupo. En ambos grupos hubo una alta mortalidad: 62,5% (10) en el grupo de hemodiálisis precoz y 41,7% (10) en el grupo de hemodiálisis tardía, siendo el uso de vasopresor (p = 0,001) el principal factor de riesgo.

**Conclusión:**

el inicio temprano de la hemodiálisis en la insuficiencia renal aguda séptica basada en las definiciones de KDIGO no influyó en el resultado. Sin embargo, el uso de vasopresor asociado a hemodiálisis en pacientes sépticos fue predictor de muerte.

## INTRODUCTION

Multiple factors may be related to the development of acute kidney injury (AKI) in critically ill patients, however the most common cause in the Intensive Care Unit (ICU) is sepsis^(1)^.

Sepsis is an organic dysfunction with high mortality, caused by a dysregulated host response to an infection, which can be aggravated by hemodynamic shock, when there is cellular circulatory dysfunction^(1)^.

Kidney Improving Global Outcomes (KDIGO) defines AKI as an abrupt decrease in glomerular filtration persisting for up to 7 days, observed by an increase in serum creatinine (SCr) and a decrease in urine output^(2)^.

The mortality of individuals with AKI is around 40% to 80%, and when associated with sepsis undergoing treatment with renal support therapy (RST), this number becomes even higher^(3,4)^. Hemodialysis (HD) is currently the RST of choice for the treatment of AKI in critically obese patients, and the prevalence of peritoneal dialysis use^(1)^ is very low.

This association (AKI and sepsis) is responsible for increased hospital stay and costs, high mortality rates and development of chronic diseases^(5)^. Today, there is no consensus on the best time to initiate HD in septic individuals; however, laboratory tests, hemodynamic instability and decision-making by the team are determining factors for its initiation^(3)^.

HD is currently considered early when performed in the first 8 hours of stage 2 diagnosis or in the first 6 hours of AKI stage 3, determined by KDIGO. HD is considered late when performed after 12 hours of progression to phase 3 of AKI, also according to KDIGO^(3)^.

Despite the correct AKI treatment, the outcome of complete renal recovery^(6)^ is still uncertain.

Currently, the Acute Dialysis Quality Initiative consensus defines reversal of renal dysfunction as the return to baseline creatinine levels and partial recovery as improvement of a patient’s condition without the need for RST^(6)^.

Thus, the present study invests efforts to identify the best moment of RST in the groups of septic patients, since most of those who survive remain with chronic kidney disease. Thus, this study aimed to analyze the influence of early HD on the outcome of septic AKI.

## METHOD

### Study Design

This is an observational, analytical, prospective study in which information about ICU patients admitted to the ICU was analyzed sequentially in a period of 6 months in 2019. The study was conducted according to the STrengthening the Reporting of Observational studies in Epidemiology (STROBE) recommendations. The research was carried out in the city of Barreiras, located in the countryside of the state of Bahia, Brazil.

### Population

The sampling process was for convenience. Patients with sepsis admitted to the ICU for more than 24 hours aged 18 years or older were included. Patients who already had chronic kidney disease before admission and those with AKI and sepsis, but without the need for RST, were excluded. The definition of sepsis used was that of the Latin American Institute of Sepsis (ILAS - *Instituto Latino Americano de Sepse*), which refers to it as a deregulated response to infection, causing an organic dysfunction^(7)^.

The presence of AKI was defined by the KDIGO criteria: increase in SCr ≥ 0.3 mg/dL in 48 hours or an increase of 1.5x in relation to the assumed baseline SCr in the last 7 days or urine output less than 0.5 mL/kg/h in 6 hours^(2)^.

The RST recommendation was performed by the nephrologist or intensivist, and there was no interference with RST recommendation, with the physicians responsible for recommendation or not of treatment.

### Data Collection

Data were collected from June 2019 to December 2019, sequentially.

To trace the sociodemographic profile, the variables sex (female and male), age (years), focus of sepsis (pulmonary origin, soft tissue, abdominal, urinary), presence of comorbidities (stroke, hypertension, diabetes, congestive heart failure), mechanical ventilation use (days), time between admission and initiation of HD (days).

The characteristics chosen for comparison between patients undergoing early and late HD were length of hospital stay and ICU length of stay (days), focus of sepsis (pulmonary, soft tissue, abdominal, urinary), mechanical ventilation (use or non-use), duration of mechanical ventilation (days), urinary output (ml/kg/h), diuresis (24 hours) on the day of admission, creatinine (mg/dl), vasopressor use and death.

The items age, sex and focus of sepsis were collected in a single moment. The other variables were collected sequentially and daily: length of hospital and ICU stay, urine output, duration of mechanical ventilation (MV), SCr, time of HD initiation (early or late) and final outcome.

The outcomes defined for this study were death (< 28 days or ≥ to 28 days), end-stage renal disease or kidney dysfunction reversal.

### Data Analysis and Treatment

For statistical analysis, the SPSS package (for Windows v.20) was used, in order to verify if the normality of data distribution was applied to the Shapiro-Wilk test.

The variables were described through their absolute and relative frequencies, and the numerical variables, through measures of central tendency and variability.

When the data normality assumption was rejected, the Kruskal-Wallis Non-Parametric Test was used, with two-by-two comparisons.

Quantitative variables were compared between groups using the Mann-Whitney U test. To verify the association between early and late HD with the outcome, the chi-square test was used. The level of significance in all tests was 5%.

### Ethical Aspects

The study was approved by the Research Ethics Committee of *Centro Universitário São Francisco de Barreiras* (UNIFASB), following the requirements of Resolution 466/2012 of the Brazilian National Health Council, in 2019, under Opinion 3,264,942. Study participants signed the Informed Consent Form (ICF) in two copies, one being given to the participant and the other being kept by the researcher.

## RESULTS

During the analysis period, 40 patients with sepsis and AKI were hospitalized, followed up until their final outcome.

The observed sociodemographic profile, as shown (Table 1), showed a mean age of 55 years and a prevalence of males (60%), being the focus of sepsis of pulmonary origin (57.5%), with a mean time of MV of 22.9 days.

**Table 1.** Sociodemographic profile of patients with septic acute kidney injury – Barreiras, BA, Brazil, 2019.

Variables	n (%) or Mean ± SD
**Sex**	
Female	16.0 (40.0)
Male	24.0 (60.0)
Age (years)	55.0 ± 16.8
**Focus of sepsis**	
Pulmonary	23.0 (57.5)
Soft tissues	4.0 (10.0)
Abdominal	11.0 (27.5)
Urinary	2.0 (5.0)
**Comorbidities**	
Stroke	10.0 (25.0)
Hypertension	29 (72.5)
Diabetes	22 (55.0)
CHF	4.0 (10.0)
MV (days)	22.9 ± 18.7
MV up to 28 days	26.0 (65.0)
Time between admission and HD (days)	1.30 ± 6.80
Death	20.0 (50.0)

CHF – congestive heart failure; MV – mechanical ventilation; HD – hemodialysis; SD – standard deviation.

No significant differences were found between the sociodemographic profile, focus of sepsis, hospital and ICU length of stay, when separating the early and late groups (Table 2).

**Table 2.** Comparative characteristics between patients undergoing early and late hemodialysis – Barreiras, BA, Brazil, 2019.

Variables	Early HD 16 (40.0%)	Late HD 24 (60.0%)	p
Length of hospital stay (days)	41.4 ± 24.3	45.3 ± 24.1	0.642*
ICU length of stay (days)	30.8 ± 19.3	29.1 ± 19.8	0.628**
**Focus of sepsis**			
Pulmonary	8.0 (50.0)	15.0 (62.5)	0.323^+^
Soft tissues	3.0 (18.7)	1.0 (4.20)	0.167^+^
Abdominal	4.0 (25.0)	7.0 (29.2)	0.533^+^
Urinary	1.0 (6.3)	1.0 (4.20)	0.646^+^
MV	16.0 (100.0)	24.0 (100.0)	–
Time under MV	22.9 ± 18.7	24.1 ± 18.9	0.081**
MV <28 days	13.0 (81.3)	13.0 (54.2)	0.079**
Urinary output at admission (ml/kg/h)	0.5 ± 0.6	0.4 ± 0.7	0.567***
Diuresis at admission (24 hours)	437.3 ± 541.1	408.9 ± 587.4	0.224***
**Creatinine value (mg/dL)**			
At recommendation	4.4 ± 1.7	4.6 ± 1.6	<0.001**
Death	10.0 (62.5)	10.0 (41.7)	0.167^+^

MV – mechanical ventilation; ICU – Intensive Care Unit; HD – hemodialysis; *Student’s t-test; **Chi-square test; ***For non-normal data (Shapiro-Wilk p <= 0.05), Mann-Whitney U test in relation to the onset of RST; ^+^Fisher’s test.

There were few days between patients’ admission to the ICU and the recommendation for HD (Table 2). Therefore, identifying the exact onset of renal injury in sepsis is still complex and its management even more complex.

The late group had a longer hospital stay as well as a greater tendency to prolonged MV (Table 2).

The difference in SCr existing between the early and late groups is related to the AKI staging itself, according to KDIGO, since, in stage 3, these values are already higher than 4 mg/dl and, therefore, an expected characteristic in this group.

Table 2 shows the difference in mean urinary output was not statistically significant between groups.

Also, there was no significant difference regarding vasopressor use (Table 3) and group outcomes (Table 4).

**Table 3.** Continuous drugs used by patients undergoing early and late hemodialysis – Barreiras, BA, Brazil, 2019.

Vasopressors	**Early HD** 16.0 (40.0%)	**Late HD** 24.0 (60.0%)	p*
Noradrenaline	13.0 (81.0)	18.0 (75.0)	
Adrenaline	1.0 (6.0)	0.0 (0.0)	
Others	2.0 (12.0)	1.0 (4.0)	0.246
None	3.0 (19.0)	6.0 (25.0)	
>1 vasopressor	2.0 (12.0)	0.0 (0.0)	
Only 1 vasopressor	11.0 (69.0)	18.0 (75.0)	

*Chi-square test; HD – hemodialysis.

**Table 4.** Distribution of outcomes of patients undergoing early and late hemodialysis – Barreiras, BA, Brazil, 2019.

Outcomes	**Early HD** 16.0 (40.0%)	**Late HD** 24.0 (60.0%)	p*
Death <28 days after admission	10.0 (63.0)	8.0 (33.0)	0.423
Death ≥28 days after admission	0.0 (0.0)	2.0 (8.5)	
End-stage kidney disease	5.0 (31.0)	12.0 (50.0)	
Kidney disease reversal	1.0 (6.0)	2.0 (8.5)	

However, when considering a new group configuration, patients who died and survived, there were no significant differences regarding sex, age, focus of sepsis, MV time, urinary output, diuresis during hospital admission and creatinine values, but there was an association between vasopressor use and death and, consequently, a shorter hospital stay (Table 5).

**Table 5.** Association between outcome of patients with acute septic kidney injury on dialysis therapy with other study variables – Barreiras, BA, Brazil, 2019.

	Outcome		p
	Death n = 20	Survival n = 20	
**Sex**			0.519**
Female	7.0 (35.0)	9.0 (45.0)	
Male	13.0 (15)	11.0 (55)	
Age (years)	58 ± 16.9	62 ± 17.2	0.356*
**Focus of sepsis**			0.323**
Pulmonary	13.0 (65.0)	10.0 (50.0)	
Soft tissues	1.0 (5.0)	3.0 (15.0)	
Abdominal	6.0 (30.0)	5.0 (25.0)	
Urinary	0.0 (0.0)	2.0 (10.0)	
MV (days)	18.7 ± 19.0	21.0 ± 18.9	0.053*
MV up to 28 days	15.0 (75)	11.0 (55)	0.185**
Length of hospital stay (days)	30.0 ± 21.3	57.0 ± 24.3	0.008*
ICU length of stay (days)	18.0 ± 14.4	37 ± 18.9	0.001*
Urinary output at admission (ml/kg/h)	0.2 ± 0.6	0.2 ± 0.7	0.187*
Diuresis at admission (24 hours)	237.5 ± 541.1	220 ± 548.0	0.589*
**Creatinine value (mg/dL)**			
At hospitalization	9.4 ± 29.3	3.0 ± 1.7	0.337*
At hemodialysis	4.1 ± 1.8	4.8 ± 1.5	0.179*
Vasopressor use	20.0 (100.0)	11.0 (55%)	0.001**
Noradrenaline	19.0 (95)	10.0 (50)	
Adrenaline	1.0 (5.0)	0.0 (0.0)	
Other	3.0 (15.0)	1.0 (5%)	
>1 vasopressor	2.0 (10)	0.0 (0.0)	
Only 1 vasopressor	15.0 (75)	11.0 (55.0)	

MV – Mechanical ventilation; ICU – Intensive Care Unit; *Student’s t-test; **Chi-square test.

## DISCUSSION

Septic AKI is a common complication in the ICU, requiring its early identification, as it is a risk factor for end-stage renal disease and death^(8)^.

The occurrence of AKI episodes contributes to the development of chronic kidney disease^(4)^. Thus, treating it in order to identify contributing factors for the reversal of dysfunction is indispensable.

About 95% of patients who remained hospitalized for more than 48 hours in the ICU developed AKI, of which 75% were associated with sepsis^(4)^.

There is a tendency to less permanence in MV by use patients who initiated HD early. In a study conducted in the city of São Paulo, the development of pulmonary congestion in non-dialysis patients with AKI was evidenced, a complication that, if not promptly treated, increases the possibility of using MV and the occurrence of infections, since the non-response to diuretics brings HD as an assertive alternative to solve the problem^(4)^.

The small difference observed in our study of urinary output and diuresis in the early and late groups was not significant to change the final outcome. However, its decrease causes water overload, with an impact on creatinine distribution volume, underestimating its value and may lead to a delay in the diagnosis of AKI and higher mortality^(9)^.

A significant difference was identified in the creatinine values between the groups under study, but it was expected, since they were separated by classification stages, and, in stage 3, creatinine is >4mg/dL.

Analysis performed comparing creatinine and survival values showed no difference in mortality between the group with low creatinine (<3 mg/dL) and the group with high creatinine (≥3 mg/dL), which suggests not being an important and determining parameter for HD initiation^(10)^.

SCr is the most used marker for the diagnosis of renal dysfunction and HD recommendation, due to its low cost and easy availability. However, it is limited, as it is influenced by several factors, such as reduced lean body mass, malnutrition, liver disease, medications, among others^(11)^.

Another important point is added, the aspect of being elevated when there is already cell death in the course of kidney injury, being questionable to assess the difficulty of a possible kidney dysfunction reversal, as a range of patients who survive septic AKI remain dependent on RST^(11)^.

It is known of other biomarkers for early detection of AKI, such as lipocalin associated with neutrophilic gelatinase (NGAL) and glomerular cystatin C, which can help reduce the delay in identifying the onset of the injury and contribute to renal recovery, but are still inaccessible in public services^(6)^.

It is also possible to analyze, in our results, the high mortality present in both groups. Septic AKI is certainly strongly associated with worse clinical outcomes. In a survey conducted among critically ill patients with AKI, sepsis caused by sepsis was associated with higher in-hospital mortality compared with AKI of any other etiology^(12)^. This high mortality makes it difficult to achieve a high sample number for research.

When analyzing a new configuration of the group of patients who died and who survived, it is noted that age, sex, focus of sepsis, duration of MV use, urinary output and diuresis did not influence the outcome, as well as when divided into early and late HD groups. In a study carried out to characterize the clinical profile and outcome of renal patients in the ICU, sex and age, as well as comorbidities, also did not impact the result, which corroborates the research^(13)^.

Furthermore, the predominance of sepsis in the pulmonary focus (71.6%) was found in other studies, such as the one carried out in Passo Fundo, RS, with 560 patients^(14)^. According to ILAS, although sepsis may be associated with any infectious focus, pulmonary is responsible for most cases^(7)^.

The high early mortality in the study group has a significant impact on the sample size and on the reduction of hospital stay^(13,14)^.

SCr, as previously mentioned, reflects the accumulation of substances not excreted by the deficiency in glomerular filtration, but it is not associated with death, since the sample profile is in dialysis therapy, managing to play the role of the kidneys. These data are in agreement with a study carried out in Novo Hamburgo, RS, in which the outcome of septic patients was assessed, and SCr did not influence the prognosis^(13)^.

Additionally, data from the study carried out in Novo Hamburgo describe that 60% of the patients used vasoactive drugs, with 94% of these progressing to death. Vasopressor use is a risk factor for mortality in the ICU, its need demonstrates hemodynamic instability in patients and, due to the drug’s mechanism of causing vasoconstriction, it is one of the causes for AKI^(13)^. These results are in line with those found in the research.

In patients with systemic inflammation such as septic patients, pathophysiological mechanisms can lead to AKI by acute tubular necrosis (ATN). Vasopressor use, although necessary, leads to intravascular volume depletion, hypotension and consequent renal hypoperfusion, resulting in prerenal AKI that, if left untreated, can also result in ATN and become classified as renal^(15)^.

Regarding nephrotoxic drugs, in a study carried out on risk factors for AKI in intensive care patients, antibiotic therapy quadrupled the chances of renal involvement. Among the main risk classes for the development of AKI, aminoglycosides, glycopeptides and polymyxins were found, however the use of noradrenaline, dopamine and dobutamine further increased the chances of participants developing AKI in nine, three and five times, respectively, demonstrating a nephrotoxicity superior to antibiotics, which have their dose corrected according to creatinine clearance and limited time of use, which is not seen during the use of vasoactive drugs^(16)^.

In our research, the onset of HD was not a decisive factor for kidney dysfunction reversal. Recently, a similar analysis showed progressively higher mortality in patients in stage 3 of the KDIGO classification in their study, which denotes a trend of association between late HD and death^(4)^.

The existence of a longer survival advantage, when early RST is initiated, was considered in previous observational studies carried out, however in a non-septic population. However, there are situations where up to 80% of the sample of patients with AKI due to sepsis did not show advantages with the early RST initiation, which corroborates the results found^(17)^.

Hemodynamic shock and AKI may infer worse survival, but a recent study showed no significant difference in 90-day mortality between patients who underwent early versus late HD^(18)^.

In another study, with patients with AKI who initiated RST early, observed in the 1-year follow-up, there was a lower occurrence of important renal events, mortality and greater renal recovery, however, in this assessment, only 21% of the renal injury was septic^(3)^.

With so many studies on RST, the heterogeneity of the criteria used to define early and late onset of RST is clear, which makes it difficult to define the best time to initiate HD^(19,20)^. Another problem found is that the study group has a high mortality rate, making it difficult to carry out research with a high sample size.

The criterion used in our study was the official one defined by KDIGO, in which the early initiation is that performed in the first 8 hours of AKI stage 2 diagnosis or 6 hours of stage 3 and, late, when HD is performed 12 hours after the progression to stage 3^(21)^.

In a recent meta-analysis, in which the moment of onset of RST was analyzed, the existence of different criteria in the definition of early and late onset was demonstrated in each of the chosen articles^(4)^.

A disadvantage in the methodology used is that the study is completely observational and, therefore, without being able to control the groups formed; however, the main strength is to evidence the discrepancy of the criteria used by physicians for AKI detection of AKI and RST recommendation in relation to its moment.

## CONCLUSION

The study demonstrated that early initiation of HD in septic AKI based on KDIGO definitions had no influence on its outcome. The high mortality in the study group and the lack of standardization in the definitions of early and late onset of HD in existing studies make it difficult to review data for standardization of conducts in the face of septic AKI.
